# A new direction in personalized medicine: multimodal joint prediction of hepatic encephalopathy risk post-TIPS

**DOI:** 10.3389/fmed.2026.1816396

**Published:** 2026-05-12

**Authors:** Lin-Feng Zhou, Hao-Huan Tang, Jian Shen, Shuai Zhang, Wan-Ci Li, Jun Tao, Xiao-Li Zhu

**Affiliations:** 1 Department of Interventional Radiology, The First Affiliated Hospital of Soochow University, Suzhou, China; 2 Department of Radiology, Hubei Cancer Hospital, Tongji Medical College, Huazhong University of Science and Technology, Wuhan, China; 3 Department of Interventional Radiology, The Affiliated Wuxi People’s Hospital of Nanjing Medical University, Wuxi, China

**Keywords:** cirrhosis, hepatic encephalopathy, machine learning, radiomics, transjugular intrahepatic portosystemic shunt

## Abstract

**Background:**

Overt hepatic encephalopathy (OHE) is a common complication after transjugular intrahepatic portosystemic shunt (TIPS), adversely affecting quality of life. This study aimed to develop a predictive model integrating manual imaging, radiomics, and clinical data to forecast OHE within 1 year post-TIPS.

**Methods:**

This retrospective study included 338 patients who underwent TIPS between November 2015 and January 2022, divided into training and validation sets (7:3). Feature selection was performed using Chi-square test, *t*-test, least absolute shrinkage and selection operator, and logistic regression. Three models were built using manual CT features (Model M), radiomics (Model R), and clinical data (Model C), respectively. A combined model (Model MRC) integrated all three. Model performance was evaluated via ROC curves, calibration plots, and decision curve analysis. The primary endpoint was OHE occurrence within 1 year post-TIPS.

**Results:**

Within 1 year after TIPS, 79 (33.4%) participants in the training group and 34 (33.3%) participants in validation group developed OHE. Three independent models and one combined model were established and evaluated in terms of their performance. The areas under the ROC curve of Model M, Model R, Model C, and Model MRC were 0.858 (95% CI: 0.809–0.907), 0.744 (95% CI: 0.681–0.808), 0.757 (95% CI: 0.692–0.821), and 0.902 (95% CI: 0.863–0.941), respectively. F1 scores were 0.861, 0.765, 0.797, and 0.891, respectively. Model MRC demonstrated superior performance compared to the other three models.

**Conclusion:**

Model MRC exhibited a considerable predictive ability for OHE within the first year after TIPS.

## 1 Introduction

Transjugular intrahepatic portosystemic shunt (TIPS) is a widely used treatment for portal hypertension, effectively leading to rapid and long-lasting symptom control (1, 2). However, the incidence of HE after TIPS is as high as 54.5%, and the presence of hepatic encephalopathy (HE) can decrease the quality of life and survival of patients, making the decision to conduct TIPS challenging (3, 4). To optimize the outcomes of these patients, it is essential to evaluate the occurrence of postoperative HE in prospective studies before the TIPS procedure.

Studies on the prediction of overt hepatic encephalopathy (OHE) after TIPS have explored various risk factors associated with OHE after TIPS. These factors can be categorized into clinical and imaging factors, such as computed tomography (CT) and magnetic resonance imaging (MRI). Clinical risk factors comprise age more than 65, diabetes, Child-Pugh score greater than 10 points, hyponatremia, increased blood creatinine levels, and sarcopenia (3). Imaging factors encompass manually measured parameters, such as anteroposterior diameter of the right lobe of the liver, maximum liver fissure diameter, and left lobe to right lobe anteroposterior diameter ratio (5). In addition, previous studies have suggested the predictive value of radiomics-based factors (6, 7), such as gray-level run length matrix (GLRLM) and gray-level co-occurrence matrix (GLCM). It has been initially indicated that radiomics features, which assess morphological changes associated with cirrhosis, can predict OHE after TIPS (5, 6, 8). The identification of additional risk factors associated with OHE after TIPS among patients with decompensated cirrhosis requires further studies. Consequently, we analyzed new clinical and CT-based imaging features to assess the risk factors of autonomous OHE following TIPS.

Predicting which patients may develop OHE after TIPS is crucial to enable preventive interventions. Through predictive mode, clinicians can identify high-risk patients before the onset of OHE symptoms, allowing for early intervention, thereby reducing the incidence and severity of OHE. However, most existing models are based on a single modality, which may limit their predictive performance and clinical applicability.

Therefore, the aim of this study was to develop and validate a multimodal predictive model integrating clinical characteristics, manually measured CT imaging features, and radiomics signatures to predict OHE after TIPS in patients with cirrhosis. By combining complementary information from different data sources, this approach may improve prediction performance compared with single-modality models and provide a useful tool for individualized risk stratification and clinical decision-making before TIPS.

## 2 Materials and methods

### 2.1 Patient selection

**FIGURE 1:**
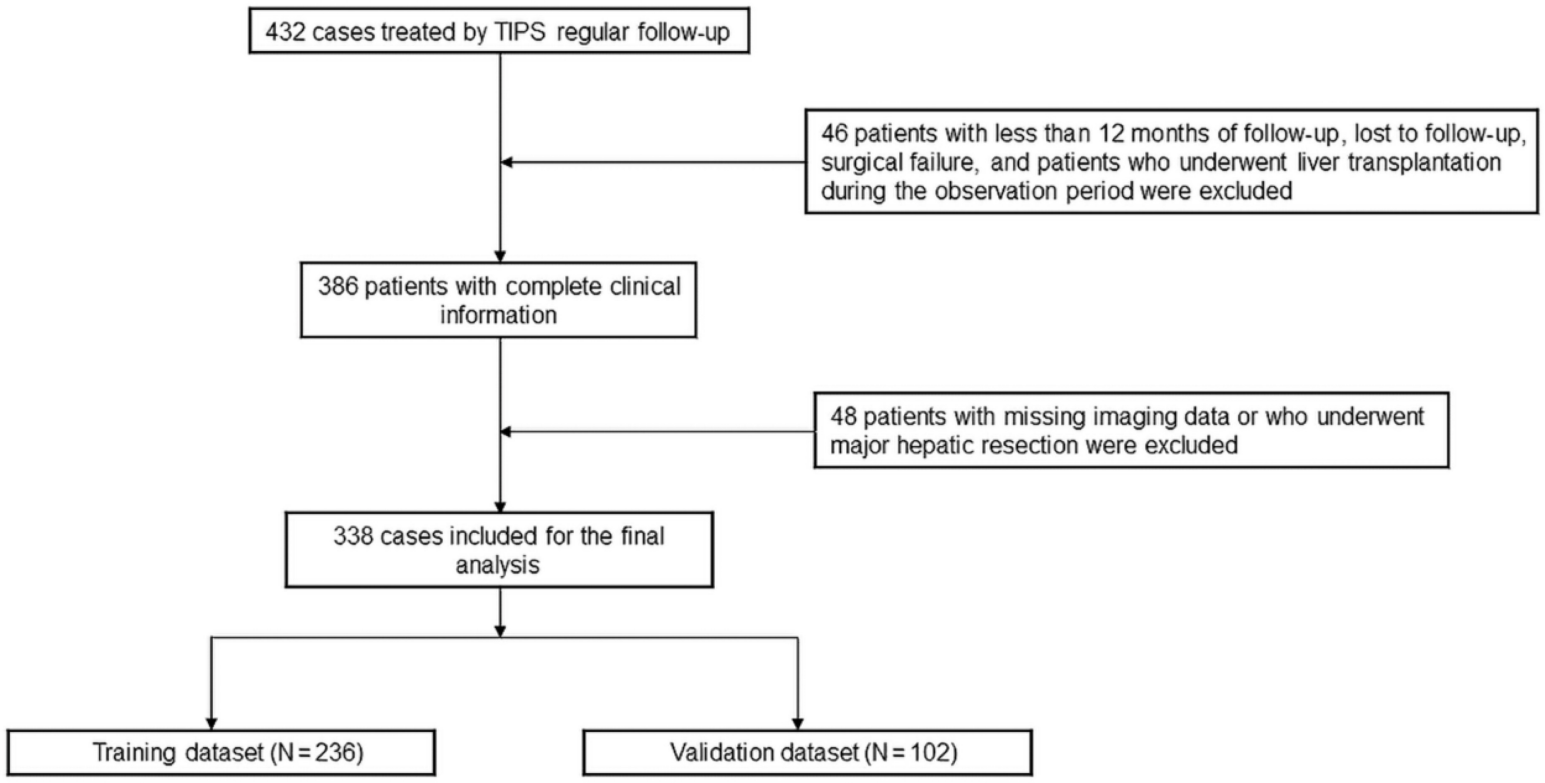
Diagram of the study design.

This study included 338 patients who underwent TIPS at the participating hospitals from November 2015 to January 2022. Before starting this study, we estimated the sample size using the 10EPV method (9). The flowchart depicting the study design is presented in Figure 1. The inclusion criteria were as follows: (1) diagnosis of portal hypertension due to liver cirrhosis; (2) intractable ascites despite conservative medical treatment or recurrent episodes of abdominal paracentesis, or recurrence of esophagogastric variceal bleeding despite treatment with medications and endoscopy, or consideration of salvage TIPS due to the high failure rate of medications and endoscopy, or considering early TIPS based on predictive risk; and (3) follow-up period of at least 12 months after the procedure. The exclusion criteria were as follows: (1) age < 18 years; (2) MELD score > 18; (3) diffuse hepatocellular carcinoma not meeting the Milan transplantation criteria (i.e., a single lesion < 5 cm or less than 3 lesions, with the largest lesion ≤ 3 cm); (4) history of major liver resection; (5) severe cardiac dysfunction; (6) patients with failed TIPS procedure, loss to follow-up, or absence of imaging data; (7) interruption of follow-up due to liver transplantation in the follow-up period. All eligible patients were randomly divided to either the training or validation sets in a 7:3 ratio. This retrospective study was conducted in accordance with the principles of the Declaration of Helsinki. Ethical approval was obtained from the Institutional Ethics Review Board of the First Affiliated Hospital of Soochow University (Approval No. 2023-235). Because the study involved only the analysis of anonymized clinical and imaging data and posed no additional risk to the patients, the requirement for written informed consent was waived by the ethics committee.

### 2.2 TIPS procedure

All patients underwent preoperative hematologic tests, including a complete blood count, liver function test, kidney function test, electrolyte level measurement, and blood ammonia level analysis. Cardiac function was evaluated through electrocardiography and echocardiography. An abdominal-enhanced CT scan was also conducted, and written informed consent was obtained. The TIPS procedures were conducted under the guidance of a digital subtracted angiography machine (Artis one or Artis zee, Siemens, Munich, Germany) by three interventional radiologists using standard techniques. Pressure sensors and monitors were used to measure the pressure of the portal vein and the central venous system during the operation. The Rups-100 suite (Cook Medical, Bloomington, IN, USA) was inserted through the jugular vein, and upon successful puncture, an 8 mm e-polytetrafluoroethylene covered stent (Viatorr; Gore, Phoenix, USA) was implanted, followed by dilation using an 8 mm balloon (Boston Scientific, Marlborough, Massachusetts, USA or Abbott, Chicago, IL, USA). Postoperatively, a portal pressure gradient less than 12 mmHg was regarded as the indicator of successful hemodynamic treatment.

### 2.3 Follow-up and outcomes

After TIPS placement, no patients received prophylactic treatment for HE, such as oral lactulose or rifaximin. Clinical baseline data and CT images were obtained within 7 days before TIPS using the medical record system. Additionally, biochemical assessments and Doppler ultrasound examinations were conducted to assess liver function and stent placement before patient discharge. Following TIPS placement, patients had monthly follow-up visits, which included telephone consultations and outpatient or inpatient visits. Patients and their families were explicitly instructed to promptly inform their physicians of any changes in the patient’s mental condition. Follow-up continued until death, liver transplantation, or the end of the study on January 31, 2023. For the primary outcome analysis, patients who underwent liver transplantation within the first year post-TIPS were excluded, as previously stated in the exclusion criteria.

The primary outcome was the incidence of OHE within 1 year after TIPS. HE was defined following the West Haven criteria (10) for grade II, III, and IV HE. Grade II is characterized by symptoms, such as lethargy, indifference, disorientation, a noticeable shift in personality, unsuitable conduct, impaired coordination, or the presence of asterixis. In grade III, patients exhibit signs of drowsiness or partial stupor, yet they can still respond to external stimuli. They may also experience confusion, significant disorientation, or atypical behavior in grade III. Grade IV is indicative of a state of coma.

### 2.4 Intra-observer and inter-observer agreement

Two technicians (Reader 1: Jian S; Reader 2: Shuai Z) conducted region of interest (ROI) segmentation, manual measurements, and radiomics feature extraction. In addition, Jian S. repeated the aforementioned procedure twice for the same patient, with a 2-weeks interval between readings, to minimize recall bias and evaluate intra-observer consistency. Inter-observer and intra-observer consistency were measured using the intraclass correlation coefficient (ICC). Data were considered valid when the inter-class ICC ≥ 0.75. The reproducibility of ROI segmentation and manual measurements was evaluated using intraclass correlation coefficients (ICC). The intra-observer ICC values ranged from 0.82 to 0.94, indicating excellent repeatability. The inter-observer ICC values ranged from 0.79 to 0.91, demonstrating good to excellent agreement between the two readers. All features included in the subsequent analysis showed ICC values greater than 0.75 and were therefore considered reliable for further modeling.

### 2.5 Clinical and radiological data collection

Candidate clinical factors predicting OHE after TIPS are presented in Table 1. Radiological features were developed and manually measured by analyzing alterations in the liver morphology (11). This approach not only builds upon findings from previous studies (5, 8), but also introduces some new characteristics. In total, we collected 24 radiological variables comprising 20 independent factors and 4 correlative factors, as follows: (1) largest aneroposterior diameter of the right lobe; (2) largest aneroposterior diameter of the left lobe; (3) maximum diameter of hepatic fissure; (4) liver transverse diameter; (5) hepatic portal vein diameter; (6) diameter of splenic vein; (7) diameter of superior mesenteric vein; (8) maximum depression of liver surface; (9) hilar periportal space (HPS); (10) signs of misalignment of right and left liver lobes (SMLL); (11) right posterior hepatic notch sign (RPS); (12) maximum gap between the outer edge of the liver and the endoperitoneum; (13) maximum depth of perihepatic fluid; (14) volume of fluid around the liver and spleen; (15) cavernous transformation of portal vein cavernous transformation (PCT); (16) liver volume (LV); (17) mean CT value of the liver; (18) liver surface area; (19) portal venous trunk velocity; (20) ratio of splenic to portal vein diameter; (21) ratio of portal vein diameter to splenic vein plus superior mesenteric vein diameter; (22) liver volume to surface area ratio; (23) ratio of anteroposterior diameter of the left lobe to the right lobe of the liver; and (24) mean CT value of lumbar vertebrae 1 to 3 (LVMCT).

**TABLE 1:** General characteristics of the included patients.

Variables	Training dataset (*N* = 236)	Validation dataset (*N* = 102)	*P*-value
Age (mean ± SD) (years)	57.2 ± 12.3	59.5 ± 12.8	0.127
Sex (Male) *n* (%)	143 (60.1%)	52 (60.0%)	0.101
Hight (m) (median [IQR])	1.7 (1.6–1.7)	1.6 (1.6–1.7)	0.176
Weight (Kg) (median [IQR])	62.2 (55.0–69.3)	61.3 (55–68)	0.248
Etiology *n* (%)			0.900
Virus	123 (52.1%)	48 (47.1%)	
Alcohol	32 (13.6%)	16 (15.7%)	
Schistosomiasis	13 (5.5%)	6 (5.9%)	
Autoimmune hepatitis	25 (10.6%)	10 (9.8%)	
Others	43 (18.2%)	22 (21.6%)	
Hypertension	49 (20.8%)	20 (19.6%)	0.925
Diabetes	66 (28.0%)	19 (18.6%)	0.093
Splenectomy	34 (14.4%)	14 (13.7%)	1.000
Stent location *n* (%)			0.645
Left branch	124 (52.5%)	51 (50.0%)	
Bifurcation	67 (28.4%)	27 (26.5%)	
Right branch	45 (19.1%)	24 (23.5%)	
CP (score) (median [IQR])	7 (6–8)	7 (6–9)	0.774
MELD (score) (median [IQR])	10 (8–12)	11 (9–13)	0.055
Ascites *n* (%)	107 (45.3%)	50 (49.0%)	0.614
Liver cancer *n* (%)	22 (9.3%)	3 (2.9%)	0.042
Laboratory parameters			
AST (U/L) (median [IQR])	31 (22–43)	32 (24–41.1)	0.806
ALT (U/L) (median [IQR])	27 (15.9–44.0)	24.5 (16.2–40.9)	0.895
PLT (10^9^/L) (median [IQR])	69 (49–108.3)	68.5 (49.3–91.0)	0.538
ALB (g/L) (mean ± SD)	31.3 ± 5.4	31.1 ± 6.0	0.845
TBIL (μmol/L) (median [IQR])	20.9 (14.2–27.7)	21.9 (16.2–29.1)	0.147
INR (median [IQR])	1.3 (1.2–1.4)	1.3 (1.2–1.5)	0.078
Cr (μmol/L) (median [IQR])	54 (63–76.5)	63.2 (51.9–73)	0.341
NH3 (μmol/L) (median [IQR])	34.8 (23.0–43.0)	34.3 (25.1–42.8)	0.912
WBC (10^9^/L) (median [IQR])	4.4 (2.7–7.2)	4.5 (2.6–7.4)	0.742
Duration of follow-up (median [IQR]) (months)	32.5 (21.8–50.0)	31.0 (20.0–48.8)	0.995

Data are expressed as median (IQR) or *n* (%). CP, Child-Pugh score; MELD, model of end-stage liver disease; AST, aspartate aminotransferase; ALT, alanine aminotransferase; PLT, platelet; ALB, albumin; TBIL, total bilirubin; INR, international normalized ratio; Cr, creatinine; NH3, blood ammonia; WBC, white blood cell.

### 2.6 ROI segmentation and feature extraction

**FIGURE 2:**
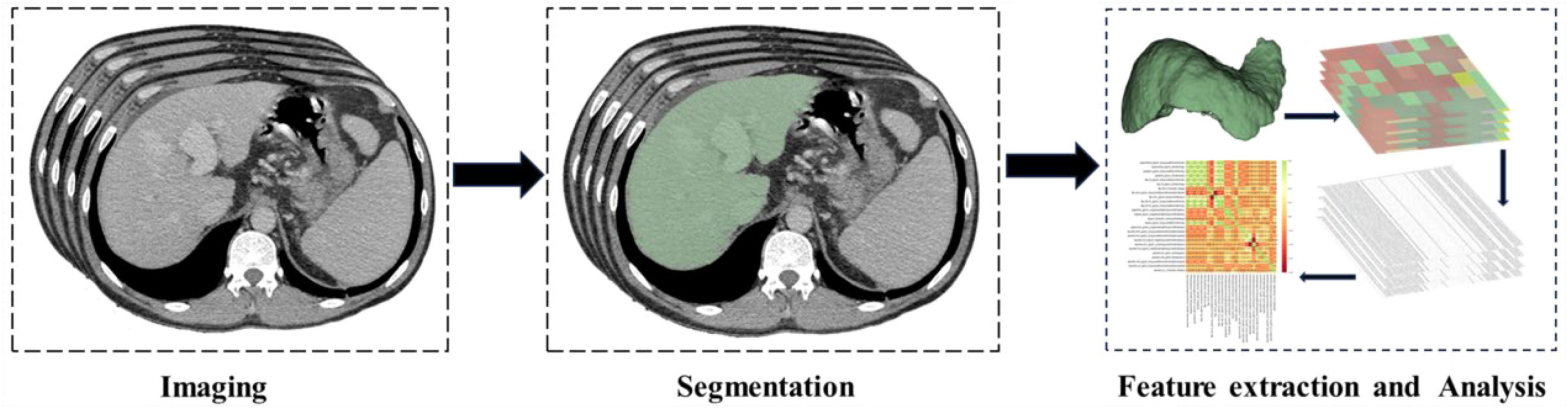
Flowchart for region of interest (ROI) segmentation, feature extraction and analysis.

We used 3D slicer software (version 5.4.0; National Institutes of Health-funded)^1^ to outline the complete liver and construct a liver model based on the portal venous phase images. We normalized the images, including N4 bias field correction (12), image normalization, and image resampling. Thereafter, we used Pyradiomics (version 3.0.1)^2^ to extract the radiomics features, including first-order statistics features, shape-based (3D) features, shape-based (2D) features, GLCM features, GLRLM features, gray-level size zone matrix features, neighboring gray-tone difference matrix features, gray-level dependence matrix features, etc. The ROI segmentation and feature extraction flowchart is shown in Figure 2.

### 2.7 Statistical analyses

Data were analyzed using R (version 4.3.1, R Foundation for Statistical Computing, Vienna, Austria) and Python (version 3.7.12, Python Software Foundation)^3^. Data following normal distribution are presented as mean ± standard deviation (mean ± SD), while data not conforming to normal distribution are represented as median and interquartile range (25th percentile, 75th percentile, IQR). The comparison between samples was conducted using either the sample *t*-test or the Wilcoxon rank-sum test. Categorical variables are expressed as percentages. They were compared using either Pearson’s chi-square test or Fisher’s exact test. For the training cohort, a normality test, *t*-test, and least absolute shrinkage and selection operator were conducted to identify the valid features and select the best subset of risk factors based on clinical and radiological factors and construct the logistic regression model (Supplementary Figures 1–3). Model discrimination was jointly assessed using the area under the curve (AUC) of the subjects’ work characteristics and the F1 score. The AUC range was interpreted as follows: 0.5–0.70 (weak discriminative ability), 0.70–0.85 (moderate discriminative ability), 0.85–0.95 (strong discriminative ability), and 0.95–1 (perfect discriminative ability). A higher F1 score indicated better model performance. Model calibration was assessed using calibration plots, and a decision curve analysis was applied to assess the usefulness of the risk model. A column-line plot of the best model was created, with statistical significance defined as *P* < 0.05.

## 3 Results

### 3.1 Patient characteristics

In total, 338 patients from two centers were included in our study, 46 patients with less than 12 months of follow-up, lost to follow-up, surgical failure and patients who underwent liver transplantation during the observation period were excluded, 48 patients with missing imaging data or who underwent major hepatic resection were excluded (Figure 1). They were randomly divided into a training cohort (236 patients with a mean age of 57.2 years and a standard deviation of 12.3) and a validation cohort (102 patients with a mean age of 59.5 years and a standard deviation of 12.8). Within 1 year of TIPS, 79 (33.4%) and 34 (33.3%) of patients in the training and validation groups developed OHE, respectively. It is worth mentioning that 22 (9.3%) patients in the training group and 3 (2.9%) patients in the validation group were complicated with liver cancer, *P* < 0.05. The baseline characteristics of the patients are presented in Table 1.

### 3.2 Construction of models

**FIGURE 3:**
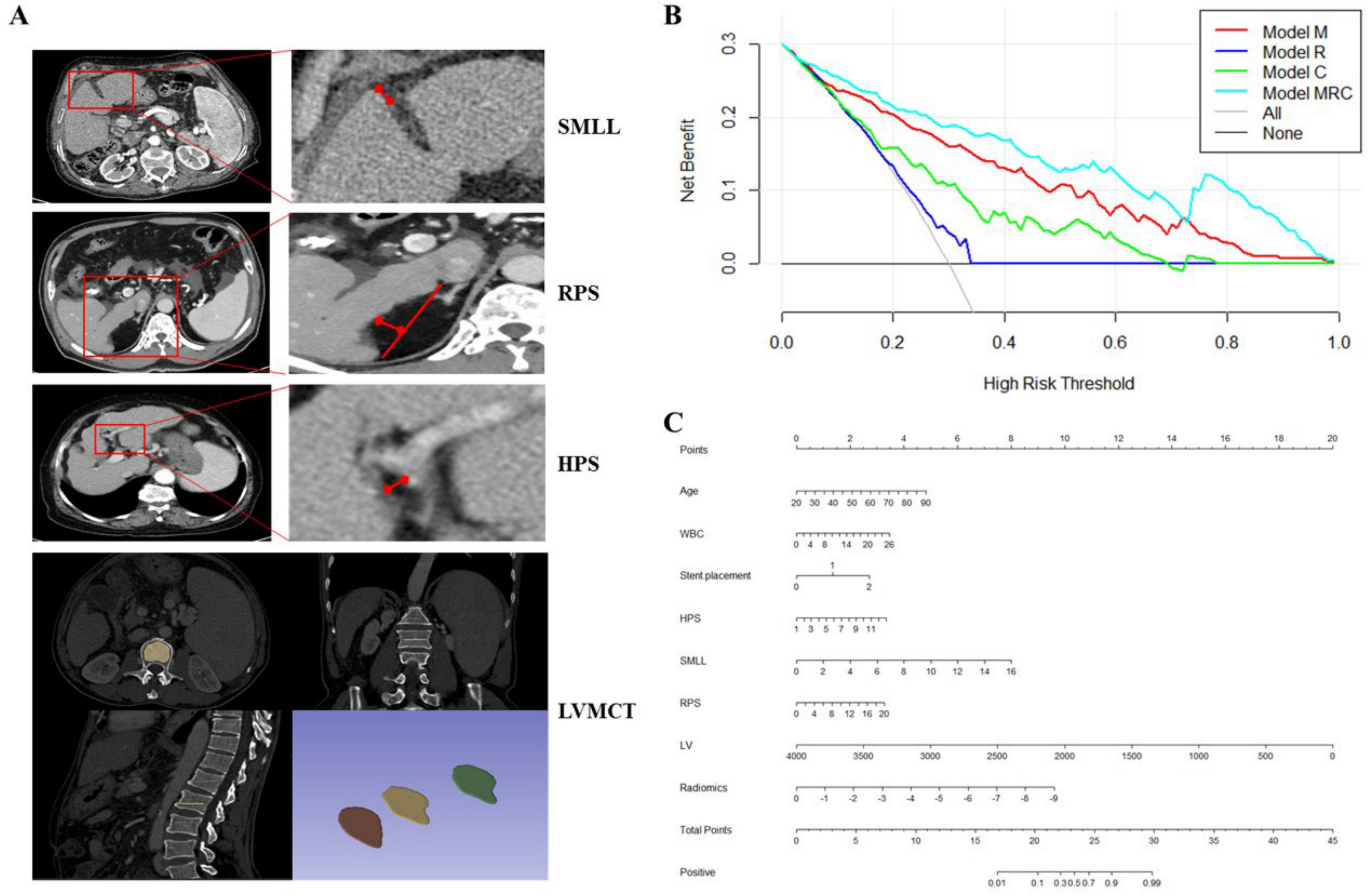
Manual measurement of CT features, decision curve and nomogram for predicting post-TIPS overt hepatic encephalopathy (OHE) based on Model MRC. **(A)** Manual measurement of CT features including signs of misalignment of right and left liver lobes (SMLL, mm), right posterior hepatic notch sign (RPS, mm), hilar periportal space (HPS, mm), measurement of CT values of cancellous bone in the 1st through 3rd lumbar vertebrae (HU) (avoiding the effects of cortical bone, degenerative structures, fractures, etc.); **(B)** decision curve analysis of the four models; **(C)** nomogram of model MRC: WBC: white blood cell, stent location (0: left branch; 1: bifurcation, 2: right branch), HPS: hilar periportal space (mm), SMLL: signs of misalignment of right and left liver lobes (mm), RPS: right posterior hepatic notch sign (mm), LV: liver volume (cm^3^).

The manual measurement-based Model (Model M) consisted of six risk features, including SMLL, RPS, HPS, LVMCT (Figure 3A), PCT, and LV. The AUCs of the training and validation sets were 0.858 and 0.869 (F1 score: 0.861). A total of 17 radiomics features were selected, mainly including first-order statistics, shape features, and texture features (e.g., GLCM, GLRLM). The full list of selected features is provided in Supplementary Table 1. The AUCs for the training and validation sets were 0.744 and 0.696, respectively (F1 score: 0.765). In terms of the usefulness of the model, we decided to adopt the model formulation of model R.


$$R\,a\,d\,i\,o\,m\,i\,c\,s\,a+b_{1}\,x_{1}+b_{2}\,x_{2}+\cdots +b_{n}\,x_{n}$$

Several features of the model R were mixed into a new feature (Radiomics) based on the above formula and used to construct the MRC model. By integrating 17 features from Model R into a single constant through logistic regression, “a” represents the constant in the logistic regression, while “b” represents the coefficient for each feature value. The magnitude of the absolute value of the coefficient reflects the weight of that feature within the model. The Clinical characterization model (Model C) consisted of five key components: height, age, white blood cell count (WBC), Child-Pugh score, and stent location. The AUC for both the training and validation datasets was 0.757, with an accompanying F1 score of 0.797.

**FIGURE 4:**
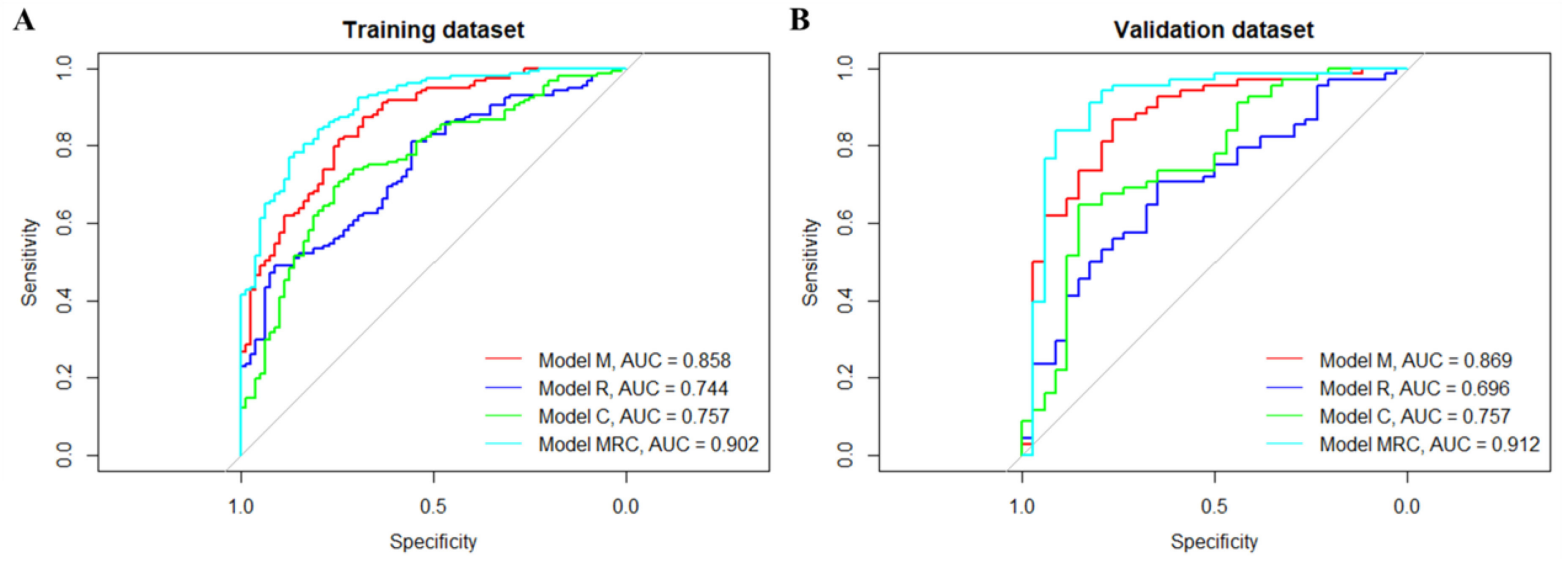
Receiver operating characteristic (ROC) curves of four models. **(A)** Training dataset roc curves; **(B)** validation dataset roc curves.

The three previously mentioned models were merged, and the feature screening model was re-executed. This culminated in the establishment of the amalgamated model (Model MRC), with an AUC score of 0.902 for the training set and 0.912 for the validation set (F1 score: 0.891). The ROC curves for all models are illustrated in Figures 4A, B, in both the training group and validation group, model MRC exhibits the best performance, followed by model M, model C, and finally model R. Table 2 elaborates on the multifactorial analysis outcomes for MRC features, 8 features were selected to construct the model, including 3 clinical features, 4 radiological features, and 1 radiomics composite feature.

**TABLE 2:** Univariate and multivariate logistic regression analysis for the combined model.

Variables	Univariate analysis		Multivariate analysis	
	OR (95% CI)	*P*-value	OR (95% CI)	*P*-value
Age	0.95 (0.93–0.98)	<0.001	0.96 (0.93–1.00)	0.043
WBC	0.94 (0.88–1.00)	0.035	0.910 (0.836–0.992)	0.033
Stent location	0.45 (0.31–0.64)	<0.001	0.38 (0.23–0.62)	<0.001
HPS	0.81 (0.69–0.94)	0.006	0.83 (0.66–1.03)	0.087
SMLL	0.73 (0.66–0.80)	<0.001	0.69 (0.61–0.79)	<0.001
RPS	0.83 (0.76–0.90)	<0.001	0.88 (0.78–1.00)	0.049
LV	1.00 (1.00–1.00)	0.004	1.00 (1.00–1.01)	<0.001
Radiomics	1.57 (1.06–2.34)	0.025	1.98 (0.98–4.00)	0.057

OR, odds ratio; CI, confidence interval; WBC, white blood cell; HPS, hilar periportal space; SMLL, signs of misalignment of right and left liver lobes; RPS, right posterior hepatic notch sign; LV, liver volume.

### 3.3 Comparison of models

We conducted a comparative analysis of the four models using their discriminant, calibration, and decision curves. Specifically, we assessed the AUC for each model on both the training and validation datasets. The AUC values for Model M, Model R, Model C, and Model MRC in the training dataset were 0.858, 0.744, 0.757, and 0.902, respectively. In the validation dataset, the AUC values for Model M, Model R, Model C, and Model MRC were 0.869, 0.696, 0.757, and 0.912, respectively (Figure 2). Both Model M and Model MRC yielded better results on calibration curves (Supplementary Figure 4). Additionally, in terms of decision curves, Model MRC demonstrated superior performance compared to Model M, Model R, and Model C (Figure 3B). Thus, we selected Model MRC as the final model and constructed the nomogram (Figure 3C).

In the development of the Model MRC, we employed a performance-oriented variable selection strategy rather than a simple *p*-value thresholding. Specifically, although HPS and the Radiomics composite feature showed *p* > 0.05 in the final multivariable analysis (Table 2), they were retained based on their contribution to the model’s overall fit and predictive power.

## 4 Discussion

In this comprehensive study, we integrated radiomics features into a conventional model to enhance the prediction of outcomes of OHE among patients with cirrhosis undergoing TIPS procedures. By combining clinical factors, manually measured imaging factors, and radiomics features using machine learning and multimodal fusion, our approach demonstrated satisfactory prediction outcomes. All four models exhibited varying levels of predictive ability. The MRC model showed the highest C-index value (0.902), suggesting its strong discrimination ability. Although the radiomics model (Model R) showed lower predictive performance compared with the manual CT-based model (Model M), it still contributed to improving the performance of the combined model. An important finding of this study is the superior predictive performance of the multimodal combined model (Model MRC). Compared with the individual models based on manual imaging features (Model M), radiomics features (Model R), or clinical variables (Model C), the MRC model achieved the highest discriminative ability, with AUC values of 0.902 in the training cohort and 0.912 in the validation cohort. This result highlights the advantage of integrating heterogeneous information from different data sources for predicting post-TIPS OHE.

This finding may be explained by the nature of the features used in each model. Manually measured CT features directly reflect macroscopic morphological changes associated with liver cirrhosis, such as liver atrophy, surface irregularity, and portal venous alterations, which are closely related to hepatic functional reserve and the development of OHE. In contrast, radiomics features capture more subtle texture heterogeneity within the liver parenchyma. While these features may provide additional microstructural information, they may not independently outperform well-established morphological indicators in this specific clinical context. Moreover, the moderate sample size and heterogeneity of the study population may also limit the standalone predictive performance of radiomics features. Although HPS and the radiomics composite feature exhibited *p* > 0.05 in the multivariable regression, they were retained to optimize the predictive architecture of Model MRC. Prioritizing predictive power over individual statistical significance resulted in a robust validation AUC of 0.912, markedly outperforming Model M (0.869) and Model C (0.757). These results suggest that while these individual features may not be independent predictors in a traditional sense, they contribute essential information that improves the model’s overall discriminative capacity and clinical utility.

Machine learning techniques have been increasingly used in healthcare information systems, particularly in the field of clinical diagnosis and prognosis assessment, like infectious diseases (13, 14). In this study, we integrated radiomics features into a conventional model for predicting the outcomes of OHE among patients with cirrhosis undergoing TIPS. This approach is commonly employed to improve prediction accuracy. Previous studies have focused on clinical and manually measured factors, in addition to separate models for radiomics features of OHE (5, 6, 8, 15). Our study integrated separate models of clinical factors, manually measured imaging factors, and radiomics features using machine learning and multimodal fusion. Consequently, we achieved satisfactory prediction outcomes.

Age, Child-Pugh score, and stent location have been identified as independent predictors of hepatic encephalopathy after TIPS (16–19). In Model C, our findings were consistent with previous studies, showing that advanced age, higher Child-Pugh score, and stent placement in the right branch of the portal vein were associated with an increased risk of OHE, while placement in the left branch was associated with a lower risk. Interestingly, our model also identified leukocyte count and height as predictors of OHE, which have not been widely reported in previous studies. Elevated leukocyte count has been associated with systemic inflammation and the severity of liver disease, suggesting a potential link with cirrhosis progression and the occurrence of OHE (20). In contrast, the role of height in OHE remains unclear. It may reflect indirect associations with body constitution or hemodynamic characteristics rather than a direct causal relationship. Therefore, this finding should be interpreted with caution and requires further investigation in future studies.

With cirrhosis progression, the liver undergoes changes in both volume and morphology. These changes include surface depressions, separation of liver lobes, and severe liver atrophy in later stages (21). The severity of cirrhosis corresponds to the liver’s ability to compensate for its functions, which is associated with the incidence of HE (22). According to this theory, Model M was developed by analyzing the liver’s morphological parameters. We discovered that HPS, SMLL, and RPS had a positive association with OHE. On the other hand, LV and LVMCT had a negative association with OHE, and PCT was shown to decrease the risk of OHE. Larger liver volumes generally indicate a better hepatic compensatory capacity (23). Furthermore, a study on TIPS procedures in patients with cirrhosis found that increased portal blood supply to the liver is positively correlated with increased hepatic volume, which may improve hepatic function (24). These findings suggest that the liver volume may be a useful marker for assessing liver function and predicting prognosis in patients with cirrhosis.

In this study, we indirectly assessed osteoporosis by measuring the CT values of cancellous bone in the L1-L3 vertebrae. The study also showed that LVMCT had good predictive efficacy. Portal vein cavernous transformation is no longer considered an absolute contraindication for TIPS (25). Our study indicated that PCT increases the risk of OHE after TIPS, even though we did not include this factor in the final Model MRC. Based on the pathophysiological characteristics of portal cavernous degeneration (26), we hypothesized that this result may be associated with the following factors: (1) Reducing portal pressure: After TIPS, most of the portal blood flow is diverted through the stent, and the tortuous collateral circulation formed by the spongy degeneration of the portal vein can help reduce the pressure of the portal vein, thereby lowering the risk of OHE; (2) Decreasing toxin entry into the brain: Portal spongiosis increases portal blood flow and reduces the likelihood of intrahepatic toxins entering the brain through the portal vein system, thereby decreasing the incidence of OHE. However, the specific mechanism of action still necessitates further studies.

Radiomics features have been shown to reflect gene expression and are associated with the staging of cirrhosis (27, 28). Liu et al. (29) constructed a non-invasive portal venous pressure model (C-index 0.849) using liver radiomics features. Wang et al. (30) constructed a radiomic model (AUC 0.90) based on liver CT and MRI to measure hepatic venous pressure gradient. This suggests that radiomics features can be used to predict HE after TIPS. A single-center retrospective study (7) included 106 patients and constructed a predictive model of HE based on radiomics, with excellent model predictive performance with an AUC of 0.899 (95% CI, 0.848–0.951) in the training group and an AUC of 0.887 (95% CI, 0.760–1.00) in the validation group. In our study, the Model R training group exhibited an AUC of 0.744 (95% CI, 0.681–0.808), while the validation group exhibited an AUC of 0.696 (95% CI, 0.590–0.803), although the standalone performance of the radiomics model was moderate, incorporating radiomics features into the combined model enhanced predictive performance. This indicates that radiomics features may offer additional complementary information beyond conventional clinical and imaging features.

The study has several limitations. First, the retrospective design could introduce some biases. For instance there may be a selection bias because we included patients from only two centers. Second, our study did not predict occult HE during follow-up because it was difficult to accurately count them. Third, although the model demonstrated good performance in the internal validation cohort, external validation in independent populations is still required to further confirm its robustness and generalizability. Future studies with larger, multicenter cohorts are warranted to validate the proposed model.

## 5 Conclusion

Our study developed a comprehensive multi-model that precisely predicts OHE after TIPS among patients with decompensated cirrhosis. Further studies are needed to confirm and enhance the credibility and usefulness of this model.

## Data Availability

The original contributions presented in this study are included in this article/Supplementary material, further inquiries can be directed to the corresponding authors.

## Footnotes

1
https://www.slicer.org

2
https://pyradiomics.readthedocs.io/en/latest/

3
https://www.python.org/

## References

[1] Rajesh S George T Philips C Ahamed R Kumbar S Mohan N et al. Transjugular intrahepatic portosystemic shunt in cirrhosis: an exhaustive critical update. *World J Gastroenterol.* (2020) 26:5561–96. 10.3748/wjg.v26.i37.5561 33088154 PMC7545393

[2] Büttner L Aigner A Pick L Brittinger J Steib C Böning G et al. 25 years of experience with transjugular intrahepatic portosystemic shunt (TIPS): changes in patient selection and procedural aspects. *Insights Imaging.* (2022) 13:73. 10.1186/s13244-022-01216-5 35416547 PMC9008097

[3] Gairing S Müller L Kloeckner R Galle P Labenz C. Review article: post-tipss hepatic encephalopathy-current knowledge and future perspectives. *Aliment Pharmacol Ther.* (2022) 55:1265–76. 10.1111/apt.16825 35181894

[4] Häussinger D Dhiman R Felipo V Görg B Jalan R Kircheis G et al. Hepatic encephalopathy. *Nat Rev Dis Primers.* (2022) 8:43. 10.1038/s41572-022-00366-6 35739133

[5] Yang Y Liang X Yang S He X Huang M Shi W et al. Preoperative prediction of overt hepatic encephalopathy caused by transjugular intrahepatic portosystemic shunt. *Eur J Radiol.* (2022) 154:110384. 10.1016/j.ejrad.2022.110384 35667296

[6] Chen X Wang T Ji Z Luo J Lv W Wang H et al. 3D automatic liver and spleen assessment in predicting overt hepatic encephalopathy before TIPS: a multi-center study. *Hepatol Int.* (2023) 17:1545–56. 10.1007/s12072-023-10570-5 37531069 PMC10661776

[7] Cheng S Yu X Chen X Jin Z Xue H Wang Z et al. CT-based radiomics model for preoperative prediction of hepatic encephalopathy after transjugular intrahepatic portosystemic shunt. *Br J Radiol.* (2022) 95:20210792. 10.1259/bjr.20210792 35019776 PMC9153699

[8] Yang Y Fu S Cao B Hao K Li Y Huang J et al. Prediction of overt hepatic encephalopathy after transjugular intrahepatic portosystemic shunt treatment: a cohort study. *Hepatol Int.* (2021) 15:730–40. 10.1007/s12072-021-10188-5 33977364 PMC8286937

[9] Peduzzi P Concato J Feinstein A Holford T. Importance of events per independent variable in proportional hazards regression analysis. II. Accuracy and precision of regression estimates. *J Clin Epidemiol.* (1995) 48:1503–10. 10.1016/0895-4356(95)00048-8 8543964

[10] Vilstrup H Amodio P Bajaj J Cordoba J Ferenci P Mullen K et al. Hepatic encephalopathy in chronic liver disease: 2014 practice guideline by the American association for the study of liver diseases and the european association for the study of the liver. *Hepatology.* (2014) 60:715–35. 10.1002/hep.27210 25042402

[11] Brancatelli G Federle M Ambrosini R Lagalla R Carriero A Midiri M et al. Cirrhosis: CT and MR imaging evaluation. *Eur J Radiol.* (2007) 61:57–69. 10.1016/j.ejrad.2006.11.003 17145154

[12] Tustison N Avants B Cook P Zheng Y Egan A Yushkevich P et al. N4ITK: improved N3 bias correction. *IEEE Trans Med Imaging.* (2010) 29:1310–20. 10.1109/TMI.2010.2046908 20378467 PMC3071855

[13] Larvie M. Machine learning in *radiology*: resistance Is Futile. *Radiology.* (2019) 290:465–6. 10.1148/radiol.2018182312 30398438

[14] Peiffer-Smadja N Rawson T Ahmad R Buchard A Georgiou P Lescure F et al. Machine learning for clinical decision support in infectious diseases: a narrative review of current applications. *Clin Microbiol Infect.* (2020) 26:584–95. 10.1016/j.cmi.2019.09.009 31539636

[15] Cao J Yang J Ming Z Wu J Yang L Chen T et al. A radiomics model of liver CT to predict risk of hepatic encephalopathy secondary to hepatitis B related cirrhosis. *Eur J Radiol.* (2020) 130:109201. 10.1016/j.ejrad.2020.109201 32738462

[16] Kloster M Ren A Shah K Alqadi M Bui J Lipnik A et al. High incidence of hepatic encephalopathy after viatorr controlled expansion transjugular intrahepatic portosystemic shunt creation. *Dig Dis Sci.* (2021) 66:4058–62. 10.1007/s10620-020-06716-2 33236314

[17] Casadaban L Parvinian A Minocha J Lakhoo J Grant C Ray C et al. Clearing the confusion over hepatic encephalopathy after TIPS creation: incidence, prognostic factors, and clinical outcomes. *Dig Dis Sci.* (2015) 60:1059–66. 10.1007/s10620-014-3391-0 25316553

[18] Butt Z Jadoon N Salaria O Mushtaq K Riaz I Shahzad A et al. Diabetes mellitus and decompensated cirrhosis: risk of hepatic encephalopathy in different age groups. *J Diabetes.* (2013) 5:449–55. 10.1111/1753-0407.12067 23731902

[19] Schindler P Seifert L Masthoff M Riegel A Köhler M Wilms C et al. TIPS modification in the management of shunt-induced hepatic encephalopathy: analysis of predictive factors and outcome with shunt modification. *J Clin Med.* (2020) 9:567. 10.3390/jcm9020567 32092979 PMC7073830

[20] Chao Y Wu P Huang J Chiu Y Lee J Chen S et al. Hepatic steatosis is associated with high white blood cell and platelet counts. *Biomedicines.* (2022) 10:892. 10.3390/biomedicines10040892 35453642 PMC9025046

[21] Yoon J Lee J Kang H Ahn S Yang H Kim E et al. Quantitative assessment of liver function by using gadoxetic acid-enhanced MRI: hepatocyte uptake ratio. *Radiology.* (2019) 290:125–33. 10.1148/radiol.2018180753 30375932

[22] Tapper E Henderson J Parikh N Ioannou G Lok A. Incidence of and risk factors for hepatic encephalopathy in a population-based cohort of Americans with cirrhosis. *Hepatol Commun.* (2019) 3:1510–9. 10.1002/hep4.1425 31701074 PMC6824059

[23] Li C Liu H Wang J Li X Cui T Wang R et al. Multiparametric MRI combined with liver volume for quantitative evaluation of liver function in patients with cirrhosis. *Diagn Interv Radiol.* (2022) 28:547–54. 10.5152/dir.2022.211325 36550754 PMC9885717

[24] He J Li J Fang C Qiao Y Feng D. The relationship and changes of liver blood supply, portal pressure gradient, and liver volume following TIPS in cirrhosis. *Can J Gastroenterol Hepatol.* (2022) 2022:7476477. 10.1155/2022/7476477 36531835 PMC9754828

[25] Tripathi D Stanley A Hayes P Travis S Armstrong M Tsochatzis E et al. Transjugular intrahepatic portosystemic stent-shunt in the management of portal hypertension. *Gut.* (2020) 69:1173–92. 10.1136/gutjnl-2019-320221 32114503 PMC7306985

[26] Attanasi M Bou Daher H Rockey D. Natural history and outcomes of cavernous transformation of the portal vein in cirrhosis. *Dig Dis Sci.* (2023) 68: 3458–66. 37349605 10.1007/s10620-023-07993-3

[27] Liu Z Wang S Dong D Wei J Fang C Zhou X et al. The applications of radiomics in precision diagnosis and treatment of oncology: opportunities and challenges. *Theranostics.* (2019) 9:1303–22. 10.7150/thno.30309 30867832 PMC6401507

[28] Lubner M Malecki K Kloke J Ganeshan B Pickhardt P. Texture analysis of the liver at MDCT for assessing hepatic fibrosis. *Abdom Radiol.* (2017) 42:2069–78. 10.1007/s00261-017-1096-5 28314916

[29] Liu F Ning Z Liu Y Liu D Tian J Luo H et al. Development and validation of a radiomics signature for clinically significant portal hypertension in cirrhosis (CHESS1701): a prospective multicenter study. *EBioMedicine.* (2018) 36:151–8. 10.1016/j.ebiom.2018.09.023 30268833 PMC6197722

[30] Wang C Huang Y Liu C Liu F Hu X Kuang X et al. Diagnosis of clinically significant portal hypertension using CT- and MRI-based vascular model. *Radiology.* (2023) 307:e221648. 10.1148/radiol.221648 36719293

