# Supplementary material for: A new direction in personalized medicine: multimodal joint prediction of hepatic encephalopathy risk post-TIPS
Source: Front Med (Lausanne). 2026 May 12;13:1816396. doi: 10.3389/fmed.2026.1816396 (PMC13201119; doi:10.3389/fmed.2026.1816396)
Supplement: Supplementary file 1 [file Table_1.docx]

**Table S1. Selected Radiomics Features for Model R**

| **No.** | **Feature Name** | **Category** |
| --- | --- | --- |
| 1 | original_firstorder_90Percentile | First-order |
| 2 | square_firstorder_90Percentile | First-order |
| 3 | wavelet.HLH_glrlm_HighGrayLevelRunEmphasis | GLRLM |
| 4 | wavelet.HLH_glrlm_LowGrayLevelRunEmphasis | GLRLM |
| 5 | wavelet.HLL_glcm_Autocorrelation | GLCM |
| 6 | exponential_glszm_ZoneEntropy | GLSZM |
| 7 | lbp.3D.k_glszm_GrayLevelNonUniformityNormalized | GLSZM |
| 8 | lbp.3D.k_glszm_GrayLevelVariance | GLSZM |
| 9 | original_shape_MinorAxisLength | Shape |
| 10 | square_firstorder_InterquartileRange | First-order |
| 11 | wavelet.HHH_glszm_HighGrayLevelZoneEmphasis | GLSZM |
| 12 | wavelet.HHH_glszm_LowGrayLevelZoneEmphasis | GLSZM |
| 13 | wavelet.HLL_glszm_GrayLevelNonUniformity | GLSZM |
| 14 | wavelet.HLL_glszm_SmallAreaLowGrayLevelEmphasis | GLSZM |
| 15 | wavelet.LHH_firstorder_Median | First-order |
| 16 | wavelet.HLL_glszm_ZonePercentage | GLSZM |
| 17 | wavelet.LHH_glrlm_RunEntropy | GLRLM |
