# Supplementary material for: A new direction in personalized medicine: multimodal joint prediction of hepatic encephalopathy risk post-TIPS
Source: Front Med (Lausanne). 2026 May 12;13:1816396. doi: 10.3389/fmed.2026.1816396 (PMC13201119; doi:10.3389/fmed.2026.1816396)
Supplement: Supplementary file 2 [file Data_Sheet_1.docx]

Supplementary Material

# Supplementary Figures

**
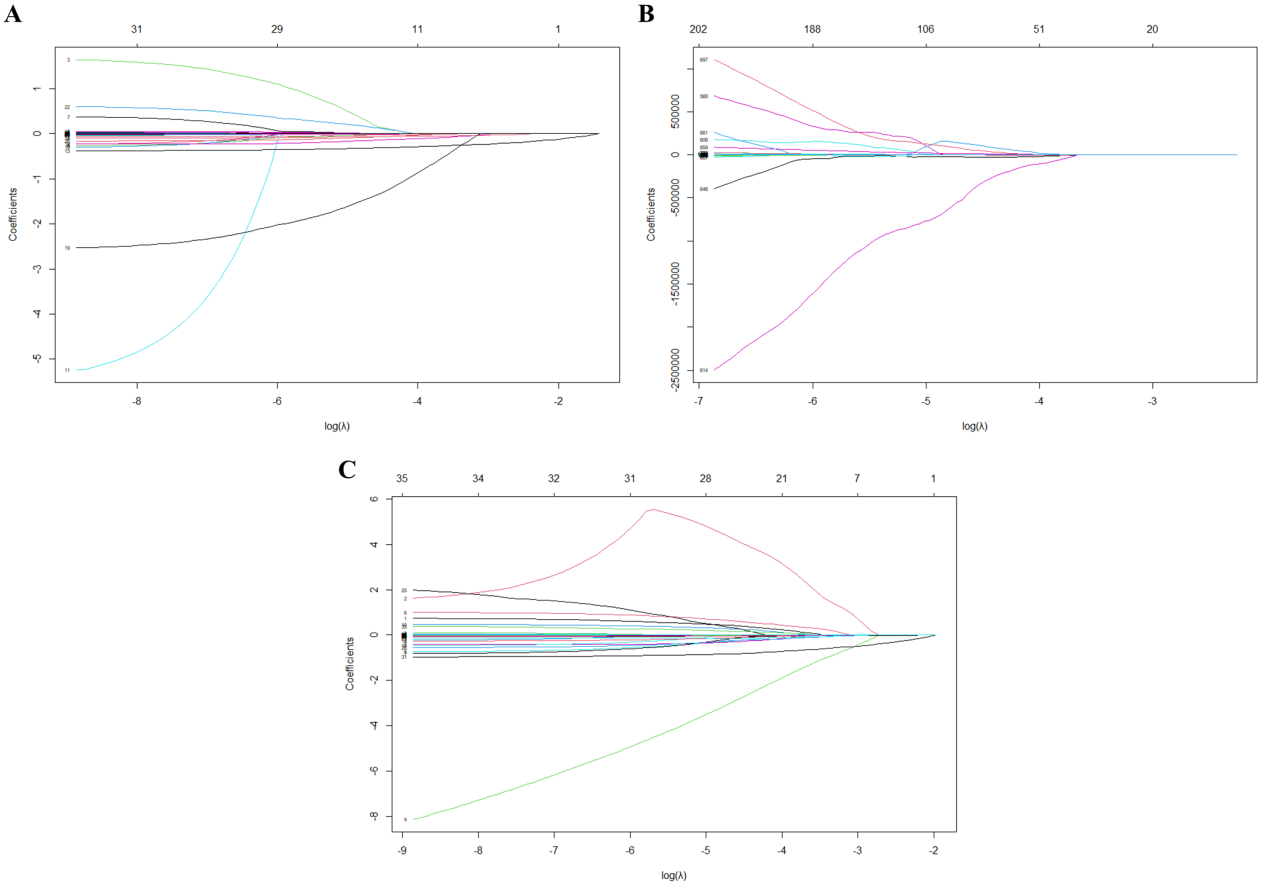
**

**Figure S1.** Regularized path map of three models. A. Regularized path map of Model M. B. Regularized path map of Model R. C. Regularized path map of Model C.

**
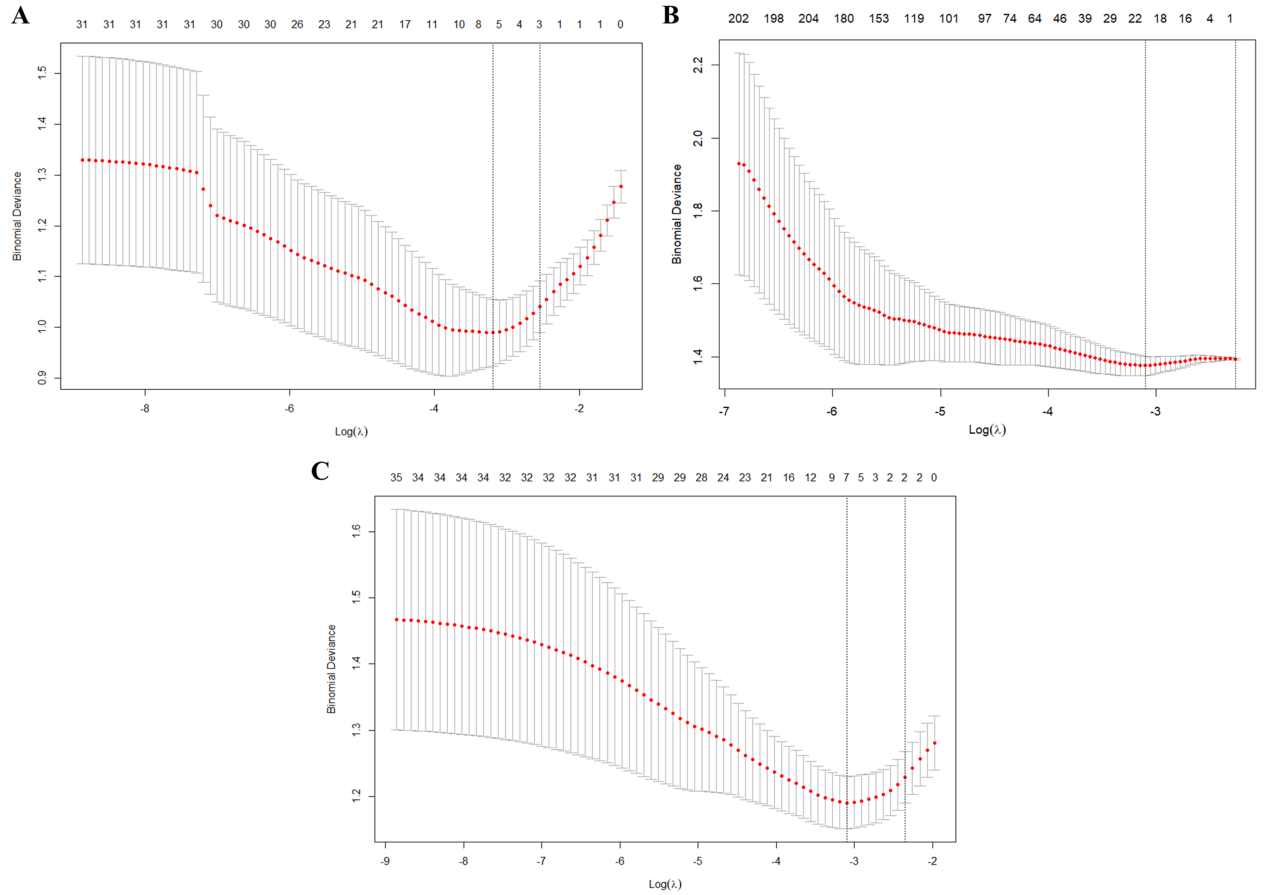
**

**Figure S2.** Cross-validation diagrams. A. Cross-validation diagram of Model M. B. Cross-validation diagram of Model R. C. Cross-validation diagram of Model C.

**
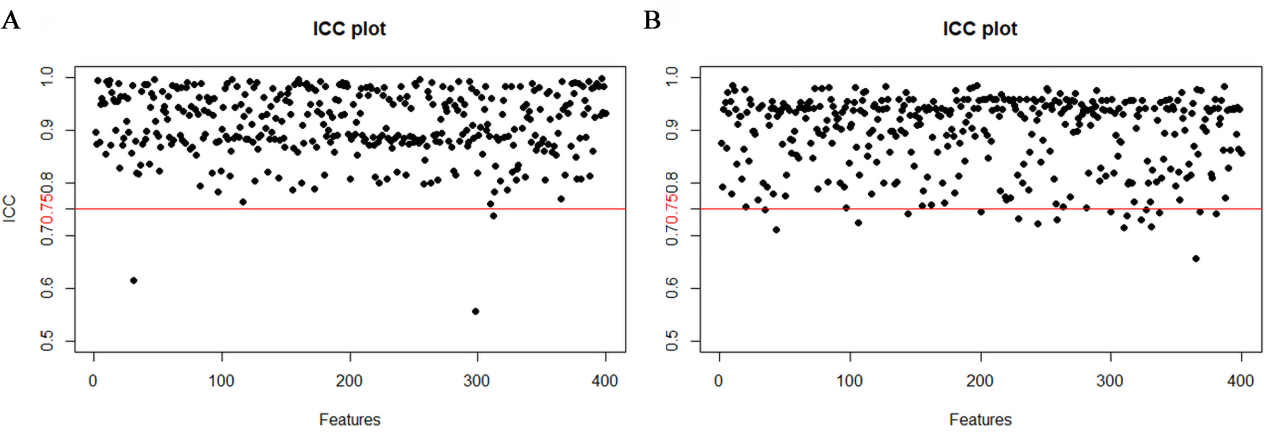
**

**Figure S3.** Intra- and inter-observer consistency diagrams. A. Intra-observer consistency diagram. B. Inter-observer consistency diagram.

**
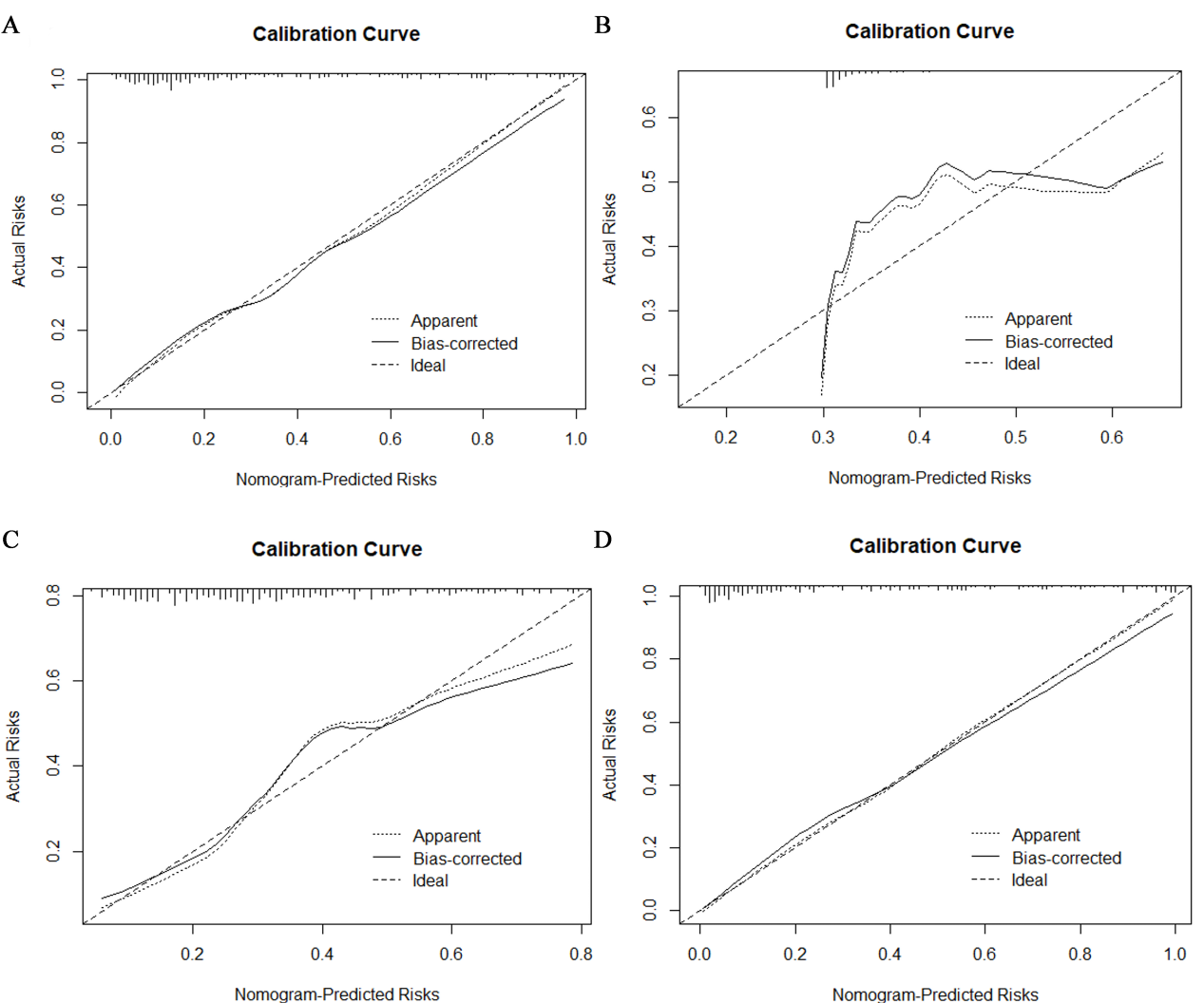
**

**Figure S4.** Calibration curves of four models. A. Calibration curve of Model M. B. Calibration curve of Model R. C. Calibration curve of Model C. D. Calibration curve of Model MRC.
